# Supplementary figures and images for: Pharmaceutical company payments to dermatology Clinical Practice Guideline authors in Japan
Source: PLoS One. 2020 Oct 13;15(10):e0239610. doi: 10.1371/journal.pone.0239610 (PMC7553305; doi:10.1371/journal.pone.0239610)

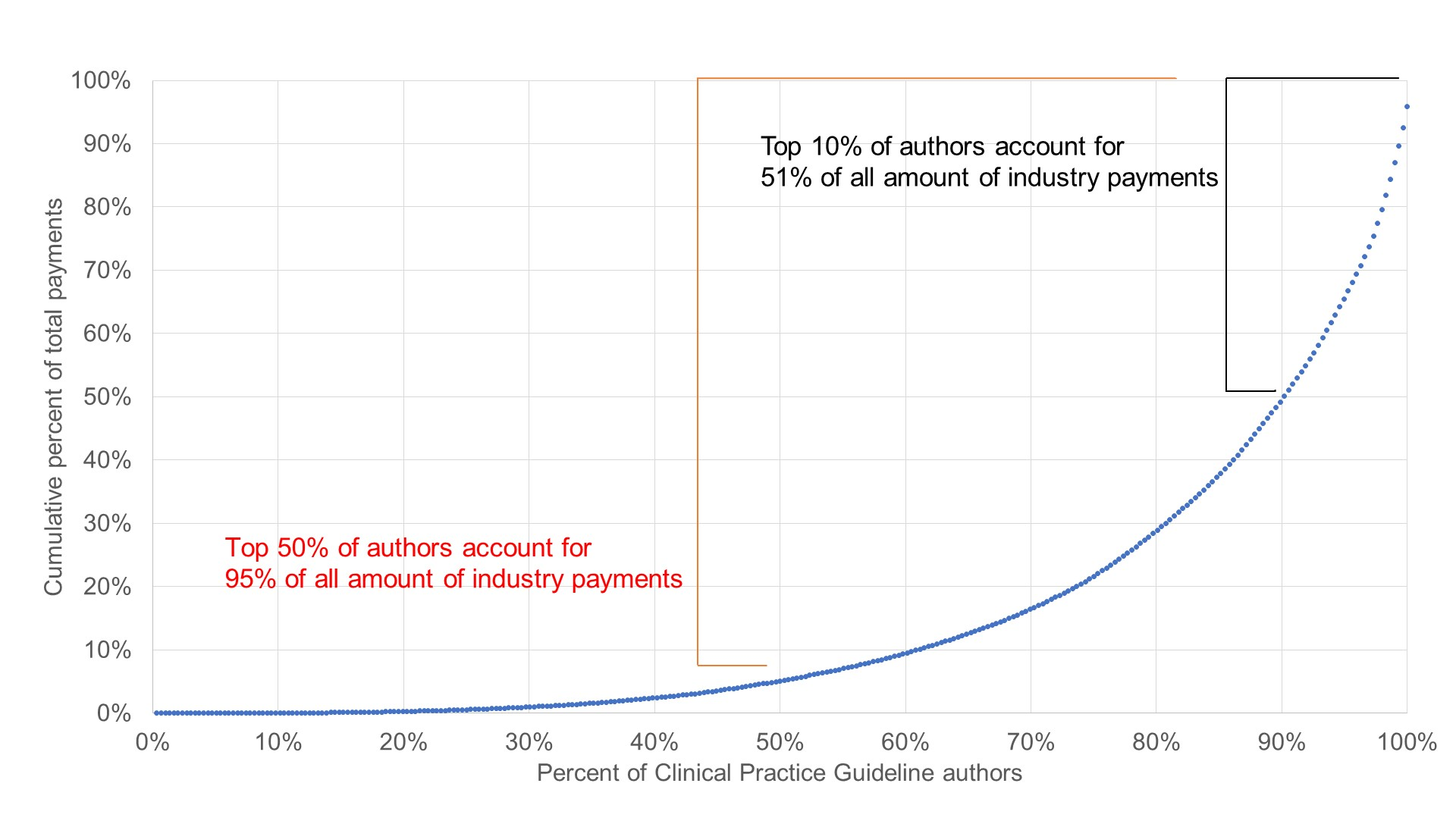

Supplement: S1 Fig — (TIF) [file pone.0239610.s001.tif]
