# Supplementary material for: Pharmaceutical company payments to dermatology Clinical Practice Guideline authors in Japan
Source: PLoS One. 2020 Oct 13;15(10):e0239610. doi: 10.1371/journal.pone.0239610 (PMC7553305; doi:10.1371/journal.pone.0239610)
Supplement: S1 Table — (DOCX) [file pone.0239610.s002.docx]

| Topics of guideline | Date of publication | Number of authors | Specially |
| --- | --- | --- | --- |
| Atopic dermatitis | November 20, 2018 | 17 | 9 dermatologists, 5 clinical immunologists, and 3 pediatricians |
| Urticaria | November 20, 2018 | 17 | 14 dermatologists and 3 clinical immunologists |
| Behçet's disease | September 20, 2018 | 6 | 6 dermatologists |
| Hand eczema | March 20, 2018 | 11 | 10 dermatologists and 1 clinical immunologist |
| Anhidrotic ectodermal dysplasia | February 20, 2018 | 9 | 7 dermatologists and 2 neurologists |
| Tuberous sclerosis complex | January 20, 2018 | 6 | 2 dermatologists, 1 pulmonologist, 1 pediatrician, 1 urologist, and 1 clinical pathologist |
| Neurofibromatosis type 1 | January 20, 2018 | 11 | 6 dermatologists, 2 orthopedic surgeons, 1 pediatrician, 1 oncologist, and 1 neurosurgeon |
| Alopecia areata | December 20, 2017 | 15 | 15 dermatologists |
| Androgenetic alopecia | December 20, 2017 | 17 | 16 dermatologists and 1 plastic surgeon |
| Pseudoxanthoma elasticum | October 20, 2017 | 17 | 11 dermatologists, 2 ophthalmologists, 2 cardiologist, 1 gastroenterologist, 1 non-physician |
| Lower leg ulcers/varicose veins | September 20, 2017 | 8 | 8 dermatologists |
| Management of burns | September 20, 2017 | 11 | 11 dermatologists |
| Pressure ulcers | August 20, 2017 | 9 | 9 dermatologists |
| Diabetic ulcer/gangrene | August 20, 2017 | 11 | 11 dermatologists |
| Skin ulcers associated with connective tissue disease/vasculitis. | August 20, 2017 | 9 | 9 dermatologists |
| Wounds in general | July 20, 2017 | 11 | 11 dermatologists |
| Bullous pemphigoid | June 20, 2017 | 16 | 15 dermatologists and 1 non-physician |
| Acne vulgaris | May 20, 2017 | 16 | 16 dermatologists |
| Vasculitis and vascular disorders | March 20, 2017 | 16 | 13 dermatologists, 2 clinical pathologists, and 1 clinical immunologist |
| Oculocutaneous albinism | February 20, 2017 | 16 | 13 dermatologists and 3 ophthalmologists |
| Lichen sclerosus  et atrophicus | November 20, 2016 | 9 | 9 dermatologists |
| Eosinophilic fasciitis | November 20, 2016 | 9 | 9 dermatologists |
| Localized scleroderma | October 20, 2016 | 9 | 9 dermatologists |
| Systemic sclerosis | September 20, 2016 | 11 | 6 dermatologists, 3 clinical immunologists, 1 cardiologist, 1 non-physician |
| Erythema exsudativum multiforme major, Stevens-Johnson syndrome and toxic epidermal necrolysis | August 20, 2016 | 29 | 24 dermatologists, 3 ophthalmologists, and 2 non-physicians |
| Generalized pustular psoriasis | November 20, 2015 | 18 | 17 dermatologists and non-physician |
| Scabies | October 20, 2015 | 22 | 17 dermatologists, 1 public health physician, and 4 non-physicians |
| Xeroderma pigmentosum | October 20, 2015 | 6 | 3 dermatologists, 1 neurologist, otolaryngologist, and orthopedic surgeon |
| The proper use of hydroxychloroquine | October 20, 2015 | 7 | 4 dermatologists, 2 clinical immunologists, and 1 ophthalmologist |
| Skin cancer | October 20, 2015 | 18 | 17 dermatologists and 1 radiologist |
| Angiosarcoma of  the face and scalp | September 20, 2015 | 10 | 8 dermatologists, 1 radiologist, and 1 non-physician |
| Primary focal hyperhidrosis | June 20, 2015 | 12 | 6 dermatologists, 4 neurologists, 1 respiratory surgeon, and 1 cardiovascular surgeon |
