# Supplementary material for: Pharmaceutical company payments to dermatology Clinical Practice Guideline authors in Japan
Source: PLoS One. 2020 Oct 13;15(10):e0239610. doi: 10.1371/journal.pone.0239610 (PMC7553305; doi:10.1371/journal.pone.0239610)
Supplement: S2 Table — Japanese yen (¥) were converted to US dollars ($) using the 2016 average monthly exchange rate of ¥108.8 per ($)1 and the 2017 average monthly exchange rate of ¥112.1 per ($)1. (DOCX) [file pone.0239610.s003.docx]

| Topics of guideline | Authors | |  | Value of Payments per Author | |  |
| --- | --- | --- | --- | --- | --- | --- |
|  | **Total,**  **No.** | **Receiving Payments**  **No. (%)** | **Total payment value** | **Median**  **(IQR)** | **Highest Payments** | |
| Hand eczema | 11 | 11 (100.0%) | ¥90,654,746  ($819,812) | ¥7,967,862  (¥4,381,218-¥12,531,476)  $72,143  ($39,505-$113,315) | ¥18,001,974  ($163,220) | |
| Systemic sclerosis | 11 | 11 (100.0%) | ¥93,168,322  ($842,967) | ¥5,999,596  (¥1,941,457-¥9,986,448)  $54,398  ($17,588-$90,227) | ¥26,545,399  ($240,651) | |
| Localized scleroderma | 9 | 9 (100.0%) | ¥71,510,404  ($646,727) | ¥5,999,596  (¥4,411,547-¥9,774,075)  $54,398  ($39,886-$88,555) | ¥21,126,909  ($191,226) | |
| Lichen sclerosus et atrophicus | 9 | 9 (100.0%) | ¥71,510,404  ($646,727) | ¥5,999,596  (¥4,411,547-¥9,774,075)  $54,398  ($39,886-$88,555) | ¥21,126,909  ($191,226) | |
| Eosinophilic fasciitis | 9 | 9 (100.0%) | ¥71,510,404  ($646,727) | ¥5,999,596  (¥4,411,547-¥9,774,075)  $54,398  ($39,886-$88,555) | ¥21,126,909  ($191,226) | |
| Atopic dermatitis | 17 | 15 (88.2%) | ¥104,760,182  ($947,775) | ¥4,576,785  (¥1,182,053-¥6,245,788)  $41,617  ($10,746-$56,406) | ¥19,810,862  ($178,593) | |
| Generalized pustular psoriasis | 18 | 17 (94.4%) | ¥72,224,960  ($653,078) | ¥3,565,008  (¥1,997,459-¥6,672,201)  $32,240  ($18,180-$60,269) | ¥10,202,291  ($92,586) | |
| The proper use of hydroxychloroquine | 7 | 7 (100.0%) | ¥26,061,380  ($236,305) | ¥3,417,631  (¥840,638-¥7,277,997)  $30,805  ($7,726-$65,947) | ¥8,789,873  ($79,931) | |
| Bullous pemphigoid | 16 | 15 (93.8%) | ¥47,443,149  ($429,172) | ¥3,313,845  (¥483,381-¥4,017,942)  $30,017  ($4,346-$36,389) | ¥11,350,039  ($102,539 | |
| Acne vulgaris | 16 | 15 (93.8%) | ¥83,295,409  ($755,303) | ¥3,243,005  (¥533,311-¥6,844,981)  $29,410  ($4,833-$61,978) | ¥32,370,286  ($293,462) | |
| Urticaria | 17 | 17 (100.0%) | ¥96,893,049  ($875,487) | ¥2,765,403  (¥1,465,636-¥6,585,081)  $25,066  ($13,228-$59,291) | ¥22,073,844  ($200,036) | |
| Skin ulcers associated with connective tissue disease/vasculitis. | 9 | 9 (100.0%) | ¥30,728,358  ($277,757) | ¥1,941,457  (¥1,030,103-¥3,852,004)  $17,588  ($9,321-$34,798) | ¥9,986,448  ($90,227) | |
| Xeroderma pigmentosum | 6 | 5 (83.3%) | ¥11,797,643  ($106,360) | ¥1,911,885  (¥367,521-¥2,789,159)  $17,277  ($3,279-$25,126) | ¥4,817,193  ($43,402) | |
| Alopecia areata | 15 | 15 (100.0%) | ¥31,079,714  ($281,029) | ¥1,661,253  (¥1,124,992-¥2,758,613)  $15,000  ($10,183-$24,862) | ¥5,287,552  ($47,796) | |
| Androgenetic alopecia | 17 | 16 (94.1%) | ¥32,534,831  ($294,272) | ¥1,627,180  (¥1,124,992-¥2,446,914)  $14,812  ($10,183-$21,985) | ¥5,287,552  ($47,796) | |
| Diabetic ulcer/gangrene | 11 | 10 (90.9%) | ¥33,950,864  ($306,895) | ¥1,470,084  (¥439,705-¥2,904,292)  $13,286  ($3,992-$26,226) | ¥12,817,586  ($115,926) | |
| Behçet's disease | 6 | 6 (100.0%) | ¥16,369,444  ($147,756) | ¥1,404,919  (¥1,030,103-¥2,144,347)  $12,717  ($9,321-$19,312) | ¥9,940,019  ($89,668) | |
| Neurofibromatosis type 1 | 11 | 11 (100.0%) | ¥37,416,848  ($337,867) | ¥1,177,187  (¥428,980-¥4,624,078)  $10,650  ($3,913-$41,499) | ¥20,687,498  ($187,147) | |
| Skin cancer | 18 | 15 (83.3%) | ¥33,708,525  ($304,579) | ¥1,044,811  (¥211,603-¥2,685,784)  $9,452  ($1,903-$24,213) | ¥7,803,278  ($70,319) | |
| Lower leg ulcers/varicose veins | 8 | 7 (87.5%) | ¥19,660,293  ($177,796) | ¥963,351  (¥174,141-¥3,699,431)  $8,693  ($1,569-$33,522) | ¥9,986,448  ($90,227) | |
| Wounds in general | 11 | 10 (90.9%) | ¥25,767,139  ($232,798) | ¥911,620  (¥236,334-¥4,085,359)  $8,178  ($2,108-$36,912) | ¥9,986,448  ($90,227) | |
| Tuberous sclerosis complex | 6 | 6 (100.0%) | ¥9,131,676  ($82,575) | ¥886,728  (¥627,904-¥1,407,332)  $8,054  ($5,671-$12,837) | ¥4,817,193  ($43,402) | |
| Vasculitis and vascular disorders | 16 | 15 (93.8%) | ¥27,161,499  ($245,851) | ¥869,669  (¥166,777-¥3,158,344)  $7,968  ($1,519-$28,495) | ¥7,277,997  ($65,947) | |
| Oculocutaneous albinism | 16 | 15 (93.8%) | ¥37,880,039  ($342,634) | ¥829,004  (¥287,220-¥3,127,171)  $7,496  ($2,611-$28,346) | ¥12,531,476  ($113,315) | |
| Anhidrotic ectodermal dysplasia | 9 | 8 (88.9%) | ¥17,175,125  ($155,181) | ¥753,521  (¥563,036-¥2,446,914)  $6,818  ($5,067-$21,985) | ¥6,644,302  ($60,048) | |
| Erythema exsudativum multiforme major, Stevens-Johnson syndrome and toxic epidermal necrolysis | 29 | 26 (89.7%) | ¥76,926,976  ($696,118) | ¥498,568  (¥111,370-¥4,635,132)  $4,527  ($993-$41,980) | ¥10,713,544  ($96,940) | |
| Pseudoxanthoma elasticum | 17 | 13 (76.5%) | ¥25,099,895  ($227,057) | ¥414,956  (¥22,274-¥1,307,117)  $3,732  ($205-$11,787) | ¥10,404,769  ($94,309) | |
| Scabies | 22 | 17 (77.3%) | ¥31,405,872  ($283,743) | ¥372,040  (¥57,434-¥1,820,913)  $3,386  ($528-$16,431) | ¥9,940,019  ($89,668) | |
| Primary focal hyperhidrosis | 12 | 11 (91.7%) | ¥26,451,254  ($239,185) | ¥333,078  (¥55,685-¥2,905,145)  $3,007  ($497-$26,288) | ¥12,531,476  ($113,315) | |
| Pressure ulcers | 9 | 9 (100.0%) | ¥15,692,911  ($141,660) | ¥315,014  (¥211,487-¥1,299,878)  $2,840  ($1,902-$11,718) | ¥9,986,448  ($90,227) | |
| Management of burns | 11 | 11 (100.0%) | ¥18,464,821  ($166,942) | ¥222,740  (¥129,519-¥2,904,292)  $2,002  ($1,190-$26,226) | ¥9,986,448  ($90,227) | |
| Angiosarcoma of the face and scalp | 10 | 9 (90.0%) | ¥4,935,917  ($44,846) | ¥183,619  (¥103,120-¥405,692)  $1,674  ($948-$3,675) | ¥2,437,902  ($22,029) | |
| Total | 296 | 269 (90.6%) | ¥788,756,266  ($7,128,762) | ¥1,136,343  (¥308,844-¥3,866,285)  $10,281  ($2,796-$34,962) | ¥32,370,286  ($293,462) | |
