# Supplementary material for: Pharmaceutical company payments to dermatology Clinical Practice Guideline authors in Japan
Source: PLoS One. 2020 Oct 13;15(10):e0239610. doi: 10.1371/journal.pone.0239610 (PMC7553305; doi:10.1371/journal.pone.0239610)
Supplement: S3 Table — (DOCX) [file pone.0239610.s004.docx]

| Year | Disclosure with individual details  No. (%) | Disclosure with an aggregated data  No. (%) | No disclosure  No. (%) |
| --- | --- | --- | --- |
| Jun 2018  ~Dec 2018 | 1 (33.3%) | 1 (33.3%) | 1 (33.3%) |
| Jan 2018  ~May 2018 | 0 (0%) | 0 (0%) | 3 (100.0%) |
| 2017 | 1 (9.1%) | 7 (63.6%) | 3 (27.3%) |
| 2016 | 0 (0%) | 0 (0%) | 4 (100.0%) |
| 2015 | 0 (0%) | 3 (60.0%) | 2 (40.0%) |
