# Supplementary material for: Pharmaceutical company payments to dermatology Clinical Practice Guideline authors in Japan
Source: PLoS One. 2020 Oct 13;15(10):e0239610. doi: 10.1371/journal.pone.0239610 (PMC7553305; doi:10.1371/journal.pone.0239610)
Supplement: S4 Table — Japanese yen (¥) were converted to US dollars ($) using the 2016 average monthly exchange rate of ¥108.8 per ($)1. (DOCX) [file pone.0239610.s005.docx]

| Category of financial conflict interest | Details of criteria |
| --- | --- |
| Lecture fee | Total annual payments of 1,000,000 JPY (9,191 USD) or more per company or organization have to be disclosed |
| Writing fee | Total annual payments of 1,000,000 JPY (9,191 USD) or more per company or organization have to be disclosed |
| Consulting fee | Total annual payments of 1,000,000 JPY (9,191 USD) or more per company or organization have to be disclosed |
| Gift and travel fee | Total annual payments of 50,000 JPY (460 USD) or more per company or organization have to be disclosed |
| Duration | Clinical practice guideline authors have to disclose payments from companies and organizations for the past 3 years before the clinical practice guideline publication. |
